# Work demands, self-care, and mental health in lawyers

**DOI:** 10.1080/13218719.2025.2497784

**Published:** 2025-05-27

**Authors:** Nora Chlap, Kristen Murray

**Affiliations:** a Master of Clinical Psychology Student, School of Medicine and Psychology, Australian National University Canberra, Australia; b School of Medicine and Psychology, Australian National University, Canberra, Australia

**Keywords:** lawyers, mental health, burnout, exhaustion, mindful self-care, passion, alcohol use, work demand

## Abstract

Lawyers face heightened risks of psychological distress, burnout, and alcohol use compared to other professions, possibly reflecting their demanding work environment and reduced engagement in protective behaviours. This study, grounded in the Job Demands-Resources theory, explored the link between workplace demands and various mental health outcomes among lawyers, as well as the role of mindful self-care, adaptive and maladaptive passion for work. One hundred and eighty-nine private practice and in-house lawyers participated in an online survey assessing work demands, mental health indicators, mindful self-care, and passion. Results indicated that increased work demands were associated with higher psychological distress and burnout, with lower levels of mindful self-care indirectly explaining these relationships. Despite prevalent alcohol use, no significant associations with work demands were evident, and passion did not act as a moderator. The findings suggest that demanding workplaces may contribute to lawyers’ distress and burnout, potentially via insufficient engagement in self-care practices.

Numerous studies have found that lawyers are disproportionately affected by psychological distress relative to other occupations and the general population (Bergin & Jimmieson, 2014; Hopkins & Gardner, 2012; Kelk et al., 2009). This is concerning given the associated consequences for lawyers, such as personal distress and unhappiness (International Bar Association, 2021), and their clients, as lawyers who are struggling with their mental wellbeing might experience impacts on their productivity, ethical judgement (International Bar Association, 2021), and capacity for empathy (Chlap & Brown, 2022). In recognition of these impacts, the literature on lawyer wellbeing has grown significantly over the past 25 years, with 104 scholarly articles published on the topic since 2000, compared to only 17 articles prior (Soon et al., 2023). However, it is important to acknowledge that the majority (58%) of the research to date comes from the United States, followed by Australia (22%), with research in other countries, such as Canada (6%) and the UK (5%), still emerging (Soon et al., 2023). Yet, there is a need for more research examining work-related demands, as well as individual characteristics, that may be common in this profession and contribute to this increased risk.

Studies consistently indicate that lawyers experience higher rates of depression, anxiety, burnout, and alcohol use than the general population. In Australia, moderate to very high levels of psychological distress were reported by 60% of respondents in a sample of 924 solicitors, with women and younger age groups reporting higher levels of distress (Kelk et al., 2009). This level of distress was almost double what is seen in the general population (Kelk et al., 2009). Furthermore, another study of 384 Australian lawyers found that 37% experienced moderate to extremely severe symptoms of depression, 31% had moderate to extremely severe symptoms of anxiety, and 49% had moderate to extremely severe stress symptoms (Bergin & Jimmieson, 2014). Similar trends have been reported in the United States, with elevated levels of stress and affective distress (e.g. anxiety, depression) detected in lawyers in numerous studies (Benjamin et al., 1990; Eaton et al., 1990; Krill et al., 2016). For example, a 2016 study of nearly 13,000 practicing lawyers found that 19%, 11%, and 14% experienced moderate to severe depression, anxiety, and stress symptoms, respectively (Krill et al., 2016). A commissioned study of 3,200 legal professionals across 124 jurisdictions by the International Bar Association in 2019 (International Bar Association, 2021) also found reduced levels of wellbeing in around half of the respondents relative to the general population, with women, younger participants, and participants from ethnic minority backgrounds reporting significantly poorer wellbeing. Interestingly, differences across work-related variables were also found, with lawyers working in-house, compared to private practice, having higher wellbeing.

Alcohol use has received comparatively less attention in research in the legal profession. However, it has been found to be three times higher in Australian lawyers than the general population (Chan et al., 2014), with as many as one in three of a sample of over 1,000 practicing Australian lawyers being classified as medium- or high-risk drinkers. These high levels of alcohol use in lawyers are also found in studies in the United States (Krill et al., 2023) and Poland (Chrobak-Kasprzyk & Jośko-Ochojska, 2020). High levels of alcohol use have also been found to be related to depression, anxiety, and stress symptoms in lawyers (Chan et al., 2014). Overall, these studies have predominantly found that more men compared to women consume alcohol at risky levels, consistent with trends in the general population (O’Brien et al., 2020). However, some studies of lawyers have found women to report higher levels of risky drinking compared to men (Anker & Krill, 2021), as well as higher levels of anxiety, depression, and stress, suggesting they might be disproportionately experiencing symptoms of mental ill-health. This higher prevalence of distress is consistent with the general population, with studies showing women generally experience symptoms of depression, anxiety, and stress at higher rates than men (Gater et al., 1998).

The evidence of risk to mental health in the legal profession has prompted efforts to understand and address this in the profession. The International Bar Association as well as professional bodies in several countries, including Australia, New Zealand, England and Wales, and the United States, have raised concerns about mental wellbeing in the legal profession. An area of focus for many of these professional bodies is the nature of workplaces and the culture within the profession, such as unsustainable working hours and excessive billing targets (International Bar Association, 2021).

The work practices of law firms are characterised by high demands and time pressure, heavy workloads, and long work hours (Chan et al., 2014). Established evidence demonstrates the consequences of these factors for mental health and wellbeing in general, with work-related psychosocial risk factors, including high job demands, low job control, and low support related to increased risk of stress-related disorders, such as anxiety, depression, and burnout (Sanne et al., 2005; Van Der Molen et al., 2020). As such, the impacts of these psychosocial aspects of work in lawyers, as well as individual characteristics of lawyers themselves which may increase their vulnerability, warrant attention in order to improve the psychological wellbeing and safety of people working in the legal profession.

A 2023 systematic review of the empirical literature found that limited existing research into lawyer wellbeing has drawn from theory, with the studies that have been grounded in theory emphasising factors such as job demands, resources, and control (Soon et al., 2023). This gap is important because theories can inform focal variables to investigate, as well as the design of interventions. One theory that can inform research into the demands associated with the legal profession and mental health is the Job-Demand Resources (JD-R) model, which states that job demands and resources play a role in workplace wellbeing (Demerouti et al., 2001). Pertinent to the current study, job demands are physical, social, or organisational aspects of the job, such as high pressure and time demands, that are associated with physical or psychological costs. For example, the presence of high demands requires sustained physical or psychological effort, leading to burnout (Demerouti et al., 2001). Extensive research has associated work demands with burnout and a causal relationship has been established in longitudinal studies (Lesener et al., 2019). The JD-R model may thus help conceptualise how the demanding work environment of lawyers creates a greater vulnerability for burnout and mental health outcomes including psychological distress and alcohol use.

Limited studies have shown that key components of the JD-R model are relevant in lawyers. For example, studies have found that burnout in lawyers is associated with high demand as well as with over-engagement with work (Nickum & Desrumaux, 2023). Additionally, stress is known to be linked with burnout, with a longitudinal study showing a causal link between stress and burnout in other professions (McManus et al., 2002). Although studies have not examined alcohol from this perspective and a meta-analysis found no evidence for longitudinal associations between job strain and alcohol intake in the general population (Heikkilä et al., 2012), higher working hours (as opposed to work stress or strain) have been related to higher levels of alcohol use, with individuals working over 48 hours a week being more likely to develop risky alcohol use as compared to individuals working 35–40 hours (Virtanen et al., 2015). It has been suggested that the high level of alcohol use in the legal profession might be due to lawyers using alcohol as a coping mechanism for high levels of stress, or that it may be ingrained into the culture of the legal profession in some jurisdictions (International Bar Association, 2021).

The JD-R model argues that one way in which job demands lead to health impairment is through the depletion of personal resources, such as an individual’s sense of their ability to control and impact their environment, as well as their self-esteem and optimism (Xanthopoulou et al., 2007). For example, some studies have found personal resources mediate relationships between work demands and emotional exhaustion (Xanthopoulou et al., 2007). One personal resource relevant to understanding the association between job demands and mental health in lawyers is *mindful self-care*. This refers to how an individual is aware of, and attends to, their internal needs and external demands by engaging in practices of self-care to address these in a way that serves one’s wellbeing and personal effectiveness (Cook-Cottone, 2019). Self-care is associated with physical health, emotional wellbeing, and mental health, and includes a range of activities that help manage stress, including engaging in sufficient rest, eating a well-balanced diet, exercising, and utilising a support system (Cook-Cottone & Guyker, 2018). Importantly, mindful self-care has been found to be related to burnout. People who practice more frequent self-care strategies report lower burnout risk (Hotchkiss & Lesher, 2018), with supportive relationships found to be especially important in preventing burnout (Kearney et al., 2009). Although mindful self-care has been investigated in occupations associated with high demands, such as doctors (Rees et al., 2020) and nurses (Slatyer et al., 2018), it has yet to be examined in the legal profession. However, as Australian lawyers generally work under conditions of high demand that require great effort, long hours, heavy workloads, and a fast pace, spill-over of work to one’s personal life is a common feature of legal practice (Chan et al., 2014). As such, it is possible that the high work demands of the legal profession impede the capacity for lawyers to engage in non-work-related activities, including self-care (Chan et al., 2014), which may explain their vulnerability to poor mental health outcomes.

Furthermore, the relationship between work demands, mindful self-care, and mental health might also be contingent on other individual factors. Although work demands and social factors have been shown to be related to burnout (Maslach et al., 2001), two individuals experiencing the same work demands in the same environment can have very different outcomes, with one thriving and the other experiencing burnout. One possible moderating variable in the professional context is an individual’s work-related attitudes, in particular, the degree and nature of passion for one’s work. Studies have distinguished between *harmonious passion*, where a person regards an activity as important to them independent of any associated contingencies (such as social acceptance) and where the person does not feel compelled to do the activity (Vallerand et al., 2003), and *obsessive passion*, where a person feels compelled to engage in an activity, due to interpersonal pressures such as social acceptance. This latter type of passion is particularly problematic, as it results in engagement with activities (such as work) with rigid persistence and in a manner outside of the person’s control, which has been associated with burnout (Vallerand et al., 2003). Although not yet considered in the legal profession, this construct has been investigated in the nursing profession, with obsessive passion found to be related to increased feelings of distress and exhaustion (Zito et al., 2022). As such, a lawyer with higher levels of obsessive passion may be particularly vulnerable to negative mental health outcomes resulting from high work demands, in part because they may be less likely to engage in self-care. Conversely, a lawyer with higher levels of harmonious passion may not engage with their work with the same level of pressure and rigidity, thus enabling maintenance of self-care and reducing their vulnerability to negative mental health outcomes.

The aim of this research is to explore the relationship between workplace demands and mental health in lawyers, and whether mindful self-care indirectly explains this relationship. Furthermore, it aims to examine the influence of obsessive and harmonious passion on these relationships, specifically whether these moderate the degree to which demands influence engagement in mindful self-care. A range of mental health indicators will be assessed in this study, specifically psychological distress, burnout, and alcohol use.

Specifically, it was hypothesised that:

**H1.** Higher workplace demands will be positively related to higher psychological distress, burnout, and alcohol use.

**H2.** Mindful self-care will indirectly explain the relationship between workplace demands and mental health and alcohol use, such that demands will be inversely related to mindful self-care, which in turn will be inversely associated with distress, burnout, and alcohol use.

**H3.** Harmonious and obsessive passion will moderate the relationship between workplace demands, mindful self-care, and mental health. Specifically, obsessive passion will strengthen the negative relationship between demands and mindful self-care, thereby increasing risk for negative outcomes, whereas harmonious passion will weaken the negative relationship between demand and mindful self-care, thereby decreasing risk for negative outcomes.

As stress and psychological distress have been shown to be greater in younger age groups (Jorm, 2000) and women (Gater et al., 1998), age and gender were controlled in the planned study analyses. Furthermore, as previous studies have found differences in mental wellbeing in lawyers who work in private practice versus those who work in-house, with in-house lawyers experiencing higher wellbeing and fewer symptoms of burnout (Chlap & Brown, 2022; International Bar Association, 2021), workplace type was also included as a covariate. Figure 1 shows the proposed full model and relationships between the variables.

**Figure 1.**
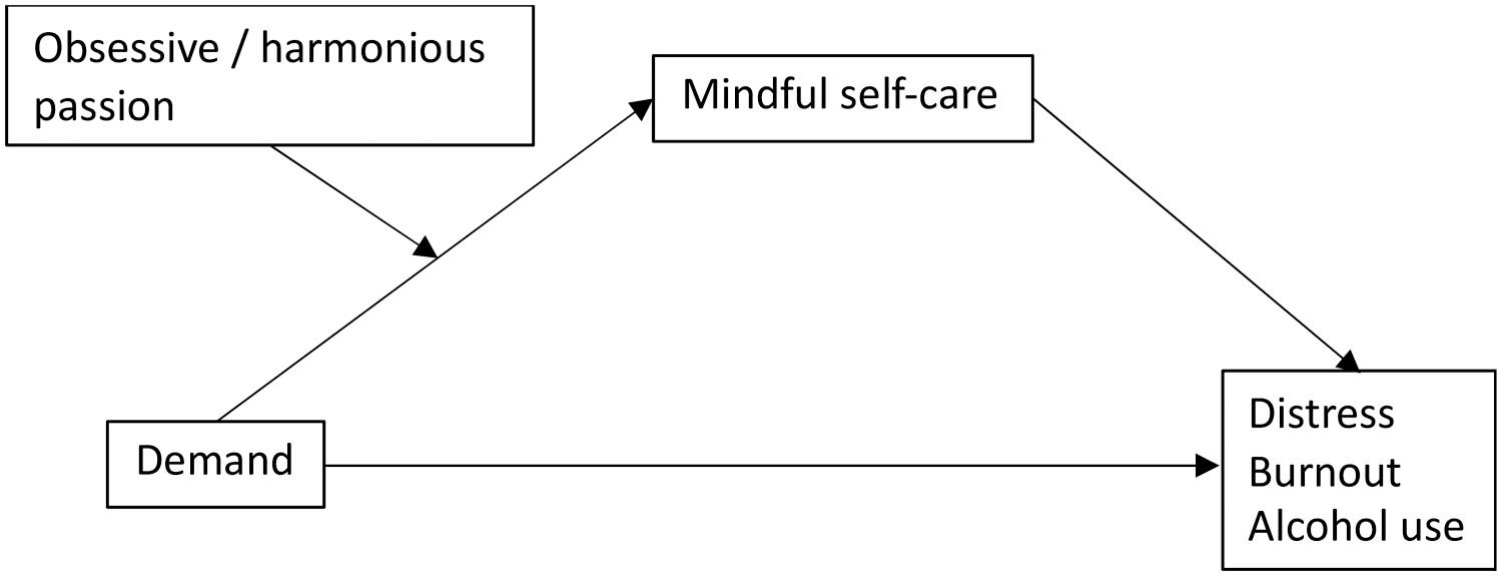
Model showing hypothesised relationship between key variables.

## Materials and methods

### Participants

Study inclusion criteria were adults aged 18 years or older and current employment as a practicing lawyer in Australia, Canada, New Zealand, the United Kingdom, or the United States. A total of 293 individuals clicked on the URL embedded in the online advertisement, of whom 205 completed the questionnaire. Sixteen people who were not practicing lawyers were excluded from analysis, leaving 189 participants, of whom 140 identified as female (74%), 47 identified as male (25%), and 2 identified as non-binary (1%). The mean age of participants was 39.2 years (*SD* = 10.07; range: 24–71 years). Demographic characteristics of the sample are provided in Table 1.

**Table 1.** Demographic characteristics of study sample (*N* = 189).

Characteristic	Number	%
Employer type		
Private practice law firm	151	79.9
Large	69	36.5
Medium	38	20.6
Small	38	20.6
In-house legal team	38	20.1
Role description/level		
Graduate or trainee lawyer	6	3.2
Junior lawyer	30	15.9
Mid-level lawyer	25	13.4
Senior associate or equivalent role in-house	65	34.4
Partner or head of legal function	53	28
‘Other’^a^	10	5.3
Country		
Australia	167	88.4%
United Kingdom	8	4.2%
North America	9	4.7%
New Zealand	4	2.1%
‘Other’	1	.5%

*Note*. ^a^Participants who selected ‘other’ were provided with a text box to describe their role, responses included ‘Special counsel’ and ‘Consultant’.

### Procedure

Ethical aspects of the study were approved by the Australian National University (ANU) Human Research Ethics Committee, Protocol #2022/621. Participants were indirectly recruited via paid advertisements placed on LinkedIn and Facebook, and via unpaid advertisements placed in Australian law society and legal industry association newsletters and websites (e.g. Law Institute of Victoria, Queensland Law Society) from November 2022 to July 2023. The paid advertisements were targeted at lawyers based in Australia, Canada, New Zealand, the United Kingdom, and the United States. Invitation emails were also sent to senior managers in large law firms and in-house legal teams in major Australian companies asking them to distribute the advertisement to their legal staff. No remuneration or benefits were offered to participants.

Interested individuals clicked on the URL embedded in the advertisement or in the email sent to them by their work manager. After reading the study information page and indicating their consent to participate, they completed the study questionnaire. Participants were informed their participation was voluntary and they could withdraw from the study at any time. Participants first completed demographic questions (age, gender, type of employer, size of law firm, years of practice, and level of seniority), and were then asked to complete questionnaires assessing workplace demands, mindful self-care, passion, alcohol use, distress, and burnout in order (questions were not randomised). Details of mental health and wellbeing resources, including emergency services, alcohol and drug use support services, and law society mental wellbeing resources were provided in both the participant information sheet and in the debrief information displayed to the participants at the conclusion of the survey. The study was delivered online using the web-based survey software Qualtrics (www.qualtrics.com). The survey took a mean of 13 minutes to complete.

### Measures

*Demographic information* was obtained, with participants asked to indicate whether they were currently employed as a lawyer; their age in years; their gender (selecting from man, woman, non-binary, not sure, prefer not to disclose, and a free text option); the type of employer they work for (selecting from private practice law firm or in-house legal team); for those employed in private practice, the type of law firm they work for (selecting from ‘large law firm employing over 500 people’; ‘medium or small law firm operating out of more than one office in Australia (for example a boutique or independent law firm with fewer than 500 employees)’; or ‘small firm or sole practitioner, operating out of a single office’, and a free text option); their years of practice as a lawyer; and role type (selecting from graduate or trainee, junior lawyer, mid-level lawyer, senior associate or equivalent, partner or head of function, and a free text option).

*Workplace demands* were assessed using the demand subscale from the 35-item HSE Management Standards Indicator Tool (Cousins et al., 2004), which measures issues such as workload, work patterns, and the work environment. This subscale contained eight items, including ‘I have unachievable deadlines’ and ‘I am pressured to work long hours’ to which participants rated their level of agreement, from 1 (never) to 5 (always), with higher scores indicating greater demand. The range of possible scores was 1–5 and the range of actual scores in this study was 1.5–5. The subscales have good internal consistency (α ranging from .78–.89; Cousins et al., 2004) and in this study Cronbach’s α for demand was .87.

*Distress* was assessed using the Distress Questionnaire 5 (DQ-5, Batterham et al., 2016), a validated brief measure used to assess psychological distress. Participants were asked to rate how much the five items applied to them over the past 30 days, from 1 (never) to 5 (always), with higher scores indicating more symptoms. The range of possible scores was 5–25 and the range of actual scores in this study was 5–25. Items included ‘In the last 30 days my worries overwhelmed me’ and ‘In the last 30 days, I felt hopeless’. A screening cut-off score of greater or equal to 11 indicates moderate current distress, where a participant is likely to meet the criteria for a wide range of disorders, whereas a DQ-5 score equal to or greater than 14 indicates high current distress, where a participant is likely to meet the criteria for specific disorders. A continuous total score was used in the models. The scale showed good internal consistency in this study (α = .85), which was in line with prior research (α = .86; Batterham et al., 2016).

*Burnout* was assessed using the Maslach Burnout Inventory (Maslach et al., 1996), a 22-item scale which measures burnout across three domains: lack of professional efficacy, emotional exhaustion, and cynicism. Only the emotional exhaustion subscale, containing seven questions, was used in the analyses to operationalise burnout, with items including ‘I feel emotionally drained by my work’. Participants were asked to indicate how often they experienced each item, using a 7-point rating scale ranging from never (0) to every day (6), with higher scores indicating greater burnout. The range of possible mean scores was 0–6 and the range of actual scores in this study was .78 to 6. The scale has good internal consistency (α = . 91; Maslach et al., 1997) and in this study it was excellent (α = .90).

*Alcohol use* was assessed using the Alcohol Use Disorders Identification Test (AUDIT; Saunders et al., 1993), which screens for heavy drinking and alcohol use disorder. The AUDIT had ten questions and responses were scored on a 5-point rating scale, ranging from 0 to 4, apart from questions 9 and 10, which asked about injury to self or others due to alcohol and whether others have expressed concern about drinking, which had possible responses of 0, 2, and 4. The range of possible scores was 0–40 and the range of actual scores in this study was 0–30. The scale showed good internal consistency in this study (α = .85). A score of 1 to 7 indicates low-risk consumption, 8–14 suggests hazardous or harmful consumption, and a score of 15 or more indicates likelihood of alcohol dependence (Saunders et al., 1993). A continuous total score was used in study models. For the purposes of this study, we use the phrase ‘problematic use’ to capture all scores indicating hazardous or harmful alcohol intake, as well as possible dependence.

*Mindful self-care* was measured using the Mindful Self-Care Scale (Cook-Cottone & Guyker, 2018). This 33-item scale measured engagement in practices including physical self-care, self-compassion and purpose, supportive relationships and structures, mindful awareness, and mindful relaxation. Items included ‘I ate a variety of nutritious foods’; ‘I took part in sports, dance or other scheduled physical activities’; and ‘I engaged in supportive and comforting self-talk’. Participants were asked to rate the frequency they engage in self-care behaviours using a 5-point rating scale ranging from never (1) to regularly (5), with higher scores indicating greater self-care (range: 1–5). Subscale scores were averaged to give a composite score, which was used in the study models, with higher scores indicating higher mindful self-care. The range of possible scores was 1–5 and the range of actual mean scores in this study was 1.39−4.03. The overall scale score showed good internal consistency in this study (α = .90).

*Passion* was assessed using the Passion Scale (Vallerand et al., 2003), which measures harmonious and obsessive passion towards work. Participants responded to each of the 14 items on a 7-point Likert scale ranging from 1 (do not agree at all) to 7 (completely agree), with higher mean scores indicating greater obsessive and harmonious passion. The range of possible and actual scores in the study was 1–7. The seven items related to obsessive passion included ‘I have difficulties controlling my urge to do my work’ and ‘I am emotionally dependent on this activity’, and the seven items related to harmonious passion included ‘This activity allows me to live a variety of experiences’ and ‘This activity is in harmony with other activities in my life’. The scales showed good to excellent internal consistency in this study, with Cronbach’s α .89 for Harmonious Passion and .93 for Obsessive Passion, which is broadly in line with previous studies which found α = .79 and α = .89 respectively (Peixoto et al., 2019). This scale has previously been used in research with other high stress professions, such as nursing (Zito et al., 2022), and was considered appropriate for this sample.

### Statistical analyses

Descriptive statistics were examined to determine the demographic features of the sample, including gender, age, employer type, role description, and country of residence. Analysis of Variance (ANOVA) examined potential differences between men and women, as well as between in-house and private practice workplaces, without making any *a priori* assertions. Associations between key variables were examined through bivariate correlations.

Hypothesis-testing employed SPSS (Version 29.1) statistical software, using the PROCESS plugin version 4.3 (Hayes, 2017). First, a set of simple mediation models was estimated using PROCESS model 4 to assess Hypotheses 1 and 2. Next, a moderated mediation model was estimated using PROCESS model 9 to assess Hypothesis 3. Age, gender, and workplace type were entered as covariates in all models. Gender and workplace were dummy coded with men being 0, women 1, private practice 0, and in-house 1 (i.e. men and private practice were the reference categories, with women and in-house the comparison groups). Percentile bootstrap confidence intervals were calculated based on 5,000 samples. Unstandardised regression coefficients (B) are reported.

An *a priori* power analysis (G*Power 3.1; Faul et al., 2009) indicated that a minimum of 155 participants were required to detect a small to medium effect size *f*^2^ = .08, with alpha set at .05, power of .95, and using up to ten predictors. For the indirect effects models, we anticipated the path from mindful self-care to burnout would be medium, based on prior studies showing a medium effect size between these variables (Hotchkiss & Lesher, 2018), but since no previous studies examining the path from demand to mindful self-care were available, this was estimated to be a medium effect size. According to Fritz and Mackinnon (2007), for a mediation analysis with a bootstrap confidence interval and medium effect sizes on both the a and b paths, a minimum sample of 78 participants is needed.

Effect size for correlations were calculated using Pearson’s *r* according to Cohen (1988); using eta^2^ for the ANOVA; and by converting R^2^ to *f*^2^ for the mediations according to Cohen (1992).

## Results

### Data screening and assumption-testing

Data screening and assumption-testing were carried out in accordance with Field (2018) and Tabachnick and Fidell (2014). The AUDIT and obsessive passion scales were positively skewed but transformed data was not used in the analyses as clinically relevant values are typically skewed (Bono et al., 2017). Assumptions of multiple linear regression were otherwise met. One univariate outlier was detected for AUDIT, but was retained as it represented clinically relevant symptoms of interest. No multivariate outliers were detected. Proportions of missing data in the current study were 0.2%. Little’s test indicated that the data (χ^2^ = 731.536, *df* = 705, *p* = .237) were missing completely at random (MCAR), with mean replacement used to replace the missing responses. Data related to the two participants who reported their gender as ‘other’ were excluded from analyses of gender differences due to insufficient power, but were retained in the overall dataset and employed in analyses where this variable was not included.

### Profile of demands, distress, alcohol use, mindful self-care, and passion in sample

Prior to model-testing, descriptive statistics and bivariate relationships between variables relevant to the aims of this study were examined and are presented in Tables 2 and 3. To characterise the distribution of primary variables in this study, mean scores were reviewed and interpreted in line with scale response options for demand, mindful selfcare, passion, and burnout, whereas comparisons with norms or clinical cut-offs for psychological distress and alcohol were used given their availability.

**Table 2.** Differences in means between female (*N* = 140) and male (*N* = 47) lawyers; and differences in means between private practice (*N* = 151) and in-house lawyers (*N* = 38) for demand, mindful self-care, passion, alcohol use, distress, and burnout (*N* = 189).

	Binary gender comparison						Workplace comparison				
	*Mean (SD)*						*Mean (SD)*				
Variable	Total (189)	Female (140)	Male (47)	*F*	*p*	*η^2^*	Private practice (151)	In-house (38)	*F*	*p*	*eta^2^*
Demand	3.48 (0.70)	3.49 (0.68)	3.48 (0.75)	.007	.935	.000	3.51 (0.70)	3.36 (0.69)	.1.437	.232	.008
Mindful self-care	2.60 (0.55)	2.60 (0.54)	2.62 (0.57)	.050	.822	.000	2.57 (0.55)	2.74 (0.51)	3.289	.071	.017
Passion											
Harmonious	4.19 (1.26)	4.25 (1.17)	3.98 (1.44)	1.706	.193	.009	4.08 (1.28)	4.63 (1.07)	6.064*	.015	.031
Obsessive	2.71 (1.47)	2.73 (1.45)	2.60 (1.55)	.295	.588	.002	2.73 (1.52)	2.61 (1.30)	.212	.646	.001
AUDIT	6.40 (5.60)	5.83 (5.50)	8.02 (6.06)	5.292*	.023	.028	6.29 (5.53)	6.86 (6.38)	.305	.582	.002
DQ5	13.85 (4.35)	14.09 (4.43)	13.12 (4.17)	1.752	.187	.009	14.04 (4.35)	13.11 (4.36)	1.390	.240	.007
Burnout	3.37 (1.33)	3.44 (1.37)	3.17 (1.19)	1.367	.244	.007	3.48 (1.31)	2.94 (1.35)	5.047*	.026	.026

*Note.* AUDIT = Alcohol Use Disorders Identification Test; DQ5 = Distress Questionnaire 5.

*p < 0.05. **p < 0.00.

**Table 3.** Correlation matrix of workplace factors, mindful self-care, passion, alcohol use, distress, and burnout.

	1	2	3	4	5	6	7	8	9
1. Age	–	.066	−.017	.001	.184*	.007	−.005	−.204**	−.126
2. Gender		–	.006	−.017	.096	.040	−.167*	.097	.086
3. Demand			–	−.374**	−.267**	.043	.053	.428**	.610**
4. Mindful self-care				–	.380**	−.056	−.039	−.349**	−.451**
5. Harmonious passion					–	.476**	−.014	−.185*	−.401**
6. Obsessive passion						–	.114	.092	−.046
7. AUDIT							–	.128	−.009
8. DQ5								–	.638**
9. Burnout									–

*Note.* AUDIT = Alcohol Use Disorders Identification Test; DQ5 = Distress Questionnaire 5.

*p < 0.05. **p < 0.001.

For primary independent variables, the mean score for demand was 3.5, indicating the participants, on average, were ‘sometimes’ to ‘often’ experiencing workloads and work patterns that they perceived as being time pressured, intense, and unachievable. The mean score for total mindful self-care was 2.6, indicating the participants, on average, engaged in self-care activities in between ‘rarely’ and ‘sometimes’. The mean score for obsessive passion was 2.7, indicating the participants, on average, ‘somewhat disagreed’ with the statements indicating obsessive passion for their work. The mean score for harmonious passion was 4.2, indicating the participants, on average, ‘neither agreed or disagreed’ with the statements indicating harmonious passion for their work.

For outcome variables in the study, the sample had a mean DQ-5 score of 13.9, which was above the cut-off of 11 indicating moderate psychological distress, and just under a score of 14, which indicates high current distress (Xu et al., 2022). Over half of the sample (55.6%) reported scores over 14. The sample mean level of distress (M = 13.8, SD = 4.35) was also significantly higher than the level of distress (M = 9.28, SD = 4.08) found in a general sample of 1,616 Australians, *t* (1803) = 14.39, *p* < .001 (Batterham et al., 2016). The mean score for burnout was 3.37, indicating that the participants, on average, were experiencing the job-related feelings indicating burnout between ‘a few times a month’ and ‘once a week’. Alcohol use in the sample was high, with a mean AUDIT score of 6.40, with over 30% of participants reporting problematic levels of drinking. This is notably higher than Australian population norms, which show that Australians on average have a mean AUDIT score of 4.58, with 22% of the population reporting problematic drinking (O’Brien et al., 2020).

#### Gender, age, and workplace differences on key variables

Examination of gender differences on primary variables could only be undertaken for participants identifying as ‘man’ or ‘woman’ due to low power in those identifying as non-binary. Analysis of Variance (ANOVA) examined potential differences between men and women, see Table 2. Results indicated that significantly more males reported problematic drinking than females, with 42.6% and 30.7% respectively. No other gender differences were evident on any other variable.

Several differences between private practice lawyers and in-house lawyers were evident, with in-house lawyers reporting significantly lower levels of burnout and significantly higher levels of harmonious passion compared to their private practice peers. No significant differences were evident on any other variable.

### Associations between demands, mental health, mindful self-care, and passion

Examination of associations between variables revealed significant positive relationships between demands, distress, and burnout, with these positive correlations being moderate and large respectively. Mindful self-care had a moderate inverse correlation with distress, burnout, and with demands. Harmonious passion had a small inverse correlation with distress, moderate inverse correlation with burnout, and small inverse correlation with demands. Obsessive passion and alcohol use did not demonstrate significant relationships with any other variables. Despite the lack of relationships between alcohol and other variables, alcohol use was retained as a dependent variable in the planned analyses. Examination of covariates indicated that younger age was associated with lower levels of harmonious passion and greater distress (both small correlations), with no other associations evident.

### Relationships between demands and mental health

A set of simple mediation analyses were conducted to test Hypothesis 1, that higher workplace demands would be positively related to distress, burnout, and alcohol use, and Hypothesis 2, that mindful self-care would indirectly explain these relationships. Separate models were performed for each of the three dependent variables: distress, burnout, and alcohol use. For each of the three outcome variables (distress, burnout, and alcohol use), demand was entered as the predictor variable, mindful self-care as the indirect effect, mental health indicator as the outcome variable, with age, gender, and workplace entered as covariates.

#### Psychological distress

**Figure 2.**
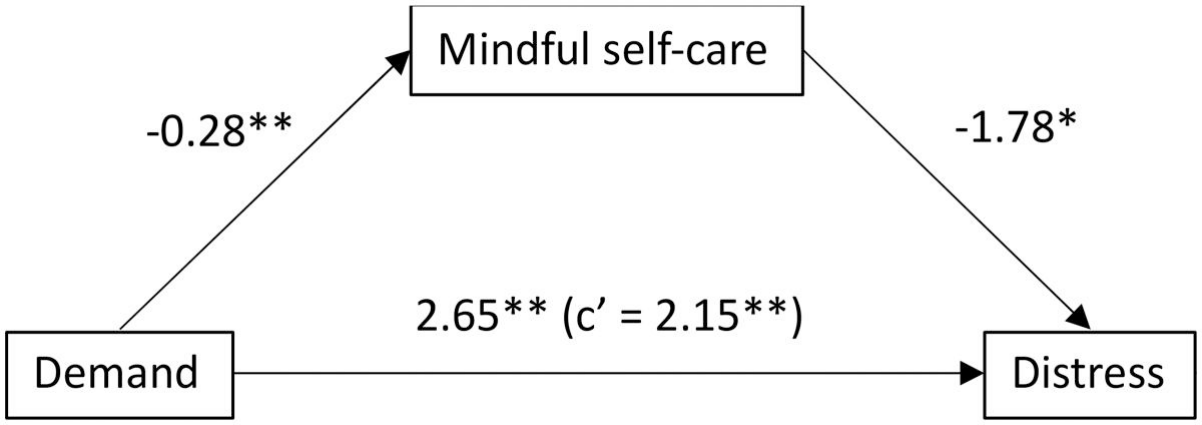
The mediating effect of mindful self-care on relationships between demand and distress. *Note.* Values are unstandardised regression coefficients. Age, gender, and workplace were included as covariates. For the final model *R*^2^ = .27, *F*(4,181) = 17.01, *p* < .001. *p<.01; **p < .001.

The total effect of demands on distress was significant, *B* = 2.65, *t*(180) = 6.46, *p* < .001, CI [1.84, 3.46], as was the direct effect, *B* = 2.15, *t*(180) = 5.00, *p* < .001, CI [1.30, 3.00], supporting Hypothesis 1. Higher demand was associated with lower mindful self-care, *B* = −0.28, *t*(180) = −5.23, *p* < .001, CI [–0.39, −0.18], and lower mindful self-care, in turn, was associated with higher distress, *B* = −1.78, *t*(179) = −3.22, *p* < .01, CI [–2.87, −0.69]. The point estimate of the indirect effect of demand on distress via mindful self-care was *B* = 0.50, SE = 0.21, with a 95% bootstrap confidence interval of between 0.15 and 0.96. As this range was entirely above zero it provided evidence of a significant positive indirect effect (*p* < .05), supporting Hypothesis 2. Hence, it can be concluded that there was an indirect association between demand and psychological distress, such that higher demand was associated with lower mindful self-care that, in turn, was associated with higher distress. The effect size for the whole model was R^2^ = .27, which converts to *f*^2^ of 0.37 and a large effect size according to Cohen (1992). A summary of the path model values is presented in Figure 2.

#### Burnout

**Figure 3.**
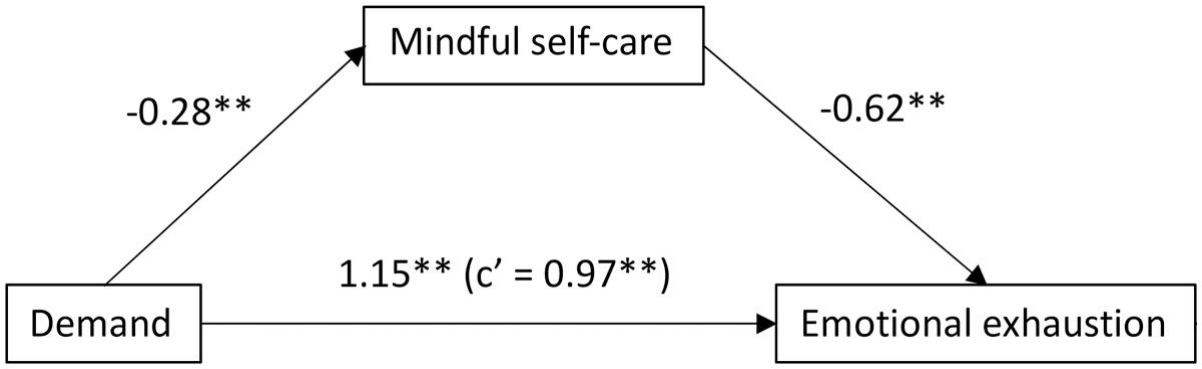
The mediating effect of mindful self-care on relationships between demand and burnout. *Note.* Values are unstandardised regression coefficients. Age, gender, and workplace were included as covariates. For the final model *R*^2^ = .45, *F*(4,181) = 37.44, *p* < .001. * *p*<.01; **p < .001.

The total effect of demands on burnout was significant, *B* = 1.15, *t*(180) = 10.43, *p* < .001, CI [0.93, 1.36], as was the direct effect, *B* = 0.97, *t*(179) = 8.62, *p* < .001, CI [0.75, 1.19], supporting Hypothesis 1. Higher demands were associated with lower mindful self-care, *B* = −0.28, *t*(180) = −5.23, *p* < .001, CI [–0.39, −0.18]. Lower mindful self-care, in turn, was associated with higher burnout, *B* = −.62, *t*(179) = −4.29, *p* < .001, CI [–0.91, −0.33]. The point estimate of the indirect effect of demand on burnout via mindful self-care was *B* = 0.18, SE = 0.06, with a 95% bootstrap confidence interval of between 0.08 and 0.30. As this range was entirely above zero it provided evidence of a significant positive indirect effect (*p* < .05), supporting Hypothesis 2. Hence, it can be concluded that there was an indirect association between demand and burnout, such that higher demand was associated with lower mindful self-care that, in turn, was associated with higher burnout. The effect size for the whole model was *R*^2^ = .45, which converts to *f*^2^ of 0.82 and is a large effect size according to Cohen (1992). A summary of the results is presented in Figure 3.

#### Alcohol use

**Figure 4.**
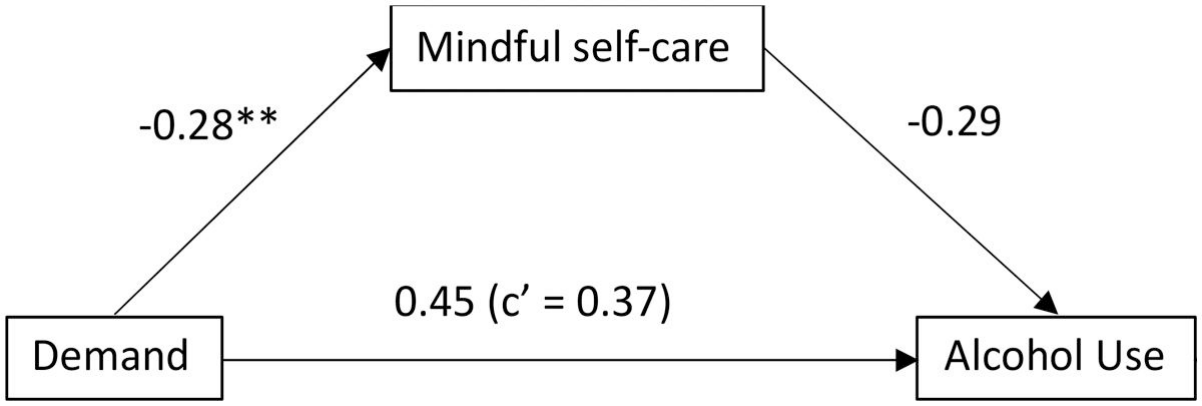
The mediating effect of mindful self-care on relationships between demand and alcohol use. *Note.* Values are unstandardised regression coefficients. Age, gender, and workplace were included as covariates. For the final model *R*^2^ = .17, *F*(4,181) = 1.31, *p* = .27. * *p*<.01; **p < .001.

The total effect of demand on alcohol use was non-significant, *B* = 0.45, *t*(180) = 0.74, *p* = .48, CI [–.74, 1.63], as was the direct effect, *B* = 0.37, *t*(179) = 0.57, *p* = .57, CI [–.91, 1.64], meaning Hypothesis 1 was not supported with regard to alcohol use. Higher demands were associated with lower mindful self-care, *B* = −0.28, *t*(180) = −5.23, *p* < .001, CI [–0.39, −0.18]. Mindful self-care, in turn, was not significantly associated with alcohol use, *B* = −.29, *t*(179) = −.35, *p* = .73, CI [–1.93, 1.35]. The point estimate of the indirect effect of demand on alcohol use via mindful self-care was *B* = 0.08, SE = 0.24, with a 95% bootstrap confidence interval of between −0.38 and 0.56. As this range included zero it did not provide evidence of a significant indirect effect (*p* < .05), therefore Hypothesis 2 in relation to alcohol use was not supported. Hence, there was not an indirect association between demand and alcohol use through mindful self-care. A summary of the results is presented in Figure 4.

### Moderating role of obsessive and harmonious passion

A moderated mediation analysis was conducted to test Hypothesis 3, that harmonious and obsessive passion would moderate the relationship between workplace demand, mindful self-care, and mental health. First, demand was entered as the predictor variable, mindful self-care as the mediating variable, obsessive and harmonious passion as the moderating variables (of the a path), distress as the outcome variable, with age, gender, and workplace entered as covariates. The interaction between demands and both harmonious passion, *B* = 0.05, *t*(176) = −0.95, *p* = .34, and obsessive passion, *B* = −0.04, *t*(176) = 1.25, *p* = .21, were non-significant. For the second moderated mediation, demand was entered as the predictor variable, mindful self-care as the mediating variable, obsessive and harmonious passion as the moderating variables, and burnout as the outcome variable, with age, gender and workplace entered as covariates. The interaction between demand and both harmonious passion and obsessive passion were both non-significant and returned the same values as the first moderated mediation (as both models shared the same a path). The indices of partial moderation are shown in Table 4. These results indicate that the indirect effect through mindful self-care is not contingent on harmonious or obsessive passion, therefore Hypothesis 3 was not supported.

**Table 4.** Moderated mediation of the relationship between demand and mindful self-care, via obsessive and harmonious passion.

Variable	Index	BootSE	BootLLCI	BootULCI
DQ5				
Harmonious passion	.0817	.0996	−.0905	.3061
Obsessive passion	−.0763	.0833	−.2603	.0741
Burnout				
Harmonious passion	.0285	.0327	−.0340	.0972
Obsessive passion	−.0266	.0286	−.0868	.0277

## Discussion

The work practices of law firms are characterised by high demands and time pressure, heavy workloads, and long work hours (Chan et al., 2014), which are known risk factors for poor mental health outcomes (Sanne et al., 2005). Numerous studies have found that lawyers are disproportionately affected by psychological distress relative to other occupations and the general population (Hopkins & Gardner, 2012), but few studies have examined the role of work demands in this increased risk across a range of mental health indicators, or the role of explanatory variables to understand these relationships. This study found high rates of poor mental health in a sample of lawyers, with support for hypotheses that demands were associated with distress and burnout, which was indirectly explained via mindful self-care. However, despite high rates in the sample, no significant associations were evident between alcohol use and work demands, nor moderation by passion for work, offering insight to wellbeing promotion in this vulnerable population.

### Profile of the sample in this study

On average, the current sample of lawyers displayed a psychosocial profile comprising moderate to high levels of workplace demands, and moderate levels of burnout and distress, with over half the sample reporting high levels of current psychological distress. Furthermore, alcohol use in the sample was high, with over 30% of participants reporting problematic levels of drinking. These findings reflect previous studies reporting higher levels of distress in the legal profession (Benjamin et al., 1990; Bergin & Jimmieson, 2014; Eaton et al., 1990; Kelk et al., 2009, Krill et al., 2016). Results are also consistent with previous studies which have found alcohol use is also higher within the legal profession, with as many as one in three lawyers being classified as high-risk drinkers (Chan et al., 2014).

Examination of individual protective and risk factors suggested that participants, on average, engaged in mindful self-care activities relatively infrequently, on average between ‘rarely’ and ‘sometimes’, which is notably lower than other studies of working professionals (Chatterjee & Jethwani, 2020). The mean level of obsessive passion was low to moderate but significantly higher than the mean level of obsessive passion in other studies and the mean level of harmonious passion was moderate and not significantly different to other professions in other studies (Zito et al, 2022).

Investigation of demographic and work-related differences also suggested some lawyers might be more vulnerable to poor mental health. Consistent with some previous studies (Chan et al., 2014, Chrobak-Kasprzyk & Jośko-Ochojska, 2020; Krill et al., 2023), significantly more men in the sample reported problematic drinking compared to women, but there were no other gender differences in contrast with previous studies which have found women lawyers to have higher levels of mental health symptoms (Anker & Krill, 2021). Furthermore, in-house lawyers had a somewhat more positive psychological profile compared to private practice lawyers, with significantly lower levels of burnout and higher levels of harmonious passion. There was no difference, however, on the level of demands, alcohol use, or distress experienced based on workplace type. Younger lawyers had a somewhat poorer psychological profile, with lower levels of harmonious passion and higher levels of distress, consistent with prior studies showing younger age is associated with higher levels of distress (Jorm, 2000; Kelk et al., 2009).

### Demands, mindful self-care, and mental health in lawyers

Hypotheses pertaining to the relationship between workplace demands and mental health in lawyers, and the indirect effect of mindful self-care in this relationship, were supported in part. Investigation of *Hypothesis 1*, that higher workplace demands would be positively related to distress, burnout, and alcohol use, indicated partial support in that positive associations were found for distress and burnout, but not alcohol use. These results are consistent with previous research showing that work demands are associated with higher levels of distress and burnout (Sanne et al., 2005; Van Der Molen et al., 2020) and suggest the pressures characteristic of the legal profession pose risk for psychological distress and burnout as has been seen in other occupational groups. This finding is also consistent with the JD-R model, which links high job demands with exhaustion. However, the results are inconsistent with previous research finding that high levels of alcohol use are related to affective distress in lawyers (Chan et al., 2014), but do align with research that has found no evidence of an association between job strain and alcohol intake (Heikkilä et al., 2012). It should be noted that the current study used a subjective measure of perceived work demand, as opposed to an objective measure of hours actually worked, which has been found to be related to higher levels of alcohol use in other studies (Virtanen et al., 2015). This may explain why the relationship between work demand and alcohol use was non-significant in this study.

Examination of *Hypothesis 2*, that associations observed between demands and mental health would be explained indirectly through mindful self-care, also revealed partial support. Indirect effects models found that higher work demands were associated with lower mindful self-care, which in turn was associated with higher distress and burnout, but not alcohol use. This finding suggests that increased work demands might deplete an individual’s personal resources, which are protective of mental wellbeing, thereby increasing a lawyer’s risk of burnout and distress. This finding is also consistent with the JD-R model, which argues that job demands lead to health impairment through the depletion of personal resources (Xanthopoulou et al., 2007).

Finally, given the importance of considering individual differences in the pathways between work demands, mindful self-care, and mental health, *Hypothesis 3* investigated whether harmonious and obsessive passion moderated focal relationships. Contrary to expectations, no significant interaction between demands and harmonious or obsessive passion was observed. Therefore, Hypothesis 3 was not supported and the results indicate that the relationship between demands and mindful self-care is not contingent on harmonious or obsessive passion. This suggests that passion (whether adaptive or maladaptive) is not influencing the relationship between work demands and mindful self-care, suggesting the reason that lawyers are more or less inclined to engage in mindful self-care is likely due to other factors.

### Implications

Taken together, these results suggest that higher work demands may increase a lawyer’s vulnerability to poorer emotional outcomes, but not higher alcohol use, when controlling for gender, age, and workplace type. Furthermore, this vulnerability might be indirectly explained via engagement in fewer mindful self-care activities, a process which was not contingent on the lawyer’s individual levels of passion for their work. As such, the study offers insight into some internal and external factors that might impact the mental health of lawyers to inform efforts to promote health and wellbeing in the legal profession. This is important in a broader context in which the legal profession has recognised and is responding to the higher vulnerability of lawyers to poorer mental health outcomes, while balancing tensions associated with the need to prioritise the wellbeing of people within an industry where revenue is contingent on lawyers billing for the time they spend working for clients. The findings suggest that reducing workplace demands may be necessary in order to enable lawyers to increase their engagement in mindful self-care activities.

The study found that the degree to which job demands influenced a lawyer’s engagement in mindful self-care was not moderated by their individual feelings of passion towards work. As such, other variables including manager support, workplace culture, and workplace demands warrant consideration. In this sample, the average level of mindful self-care was relatively low, with the average level of frequency of engaging in self-care activities being between ‘rarely’ and ‘sometimes’. These findings suggest that the reasons a lawyer does not engage in mindful self-care may be outside of their individual control, and future studies should examine whether the impediment is practical or attitudinal. Due to the relatively constrained range (i.e. low levels) of mindful self-care in this sample, future research could examine whether specific aspects of self-care have more or less of a relationship with mental health outcomes.

Alcohol use stood out as a particularly interesting finding of this study. Alcohol use in the sample was high, with over 30% of the participants reporting problematic levels of drinking. Of note, these levels appeared to be higher than reported Australian population norms (O’Brien et al., 2020) and are also consistent with previous studies of alcohol use in lawyers (Chan et al., 2014). Male lawyers in particular had high levels of problematic alcohol use, though levels of alcohol use amongst female lawyers was also higher than population norms. Despite the frequency of alcohol use in the sample, there was, however, no correlational relationship between alcohol use and any other variable in the study, and there was no relationship between work demands and alcohol use. This suggests that the high level of alcohol use in the legal industry might not, at least in this sample, be related to the demanding work environment. The lack of association between alcohol and the other variables suggests other factors are at play, for example, cultural and social norms within the profession, as has been suggested in other studies (International Bar Association, 2021). It is also possible that alcohol use is a maladaptive coping strategy, which should be considered in future studies. Given the established impacts of alcohol on mental and physical health, further investigation of alcohol use in the legal profession is needed.

The findings provide further support for the JD-R model (Demerouti et al., 2001), as higher levels of job demands were associated with higher levels of distress and burnout, with this pathway indirectly explained through a personal resource, mindful self-care. The findings additionally support the need for profession-specific studies and adaptations of theories, as profession-specific differences exist (in this case, the high use of alcohol in the sample appears to be a cultural feature of the legal profession). Future studies could additionally examine organisational specific variables and resources, such as support.

### Strengths, limitations, and future directions

This study had several strengths, including collecting a sufficiently large sample of a time poor (and therefore difficult to reach) population. It was also the first known study to examine a broader conceptualisation of ‘wellbeing’ in the legal profession, incorporating elements of physical self-care and social wellbeing (by assessing mindful self-care). This study drew from the Job Demands-Resources model of stress, and utilised a profession-specific model to consider indirect effects and moderators that were informed by unique characteristics of the focal population.

The results of this study should be interpreted considering several study limitations. First, self-report measures of demands, distress, emotional exhaustion, alcohol use, mindful self-care, and passion were used, rather than clinical interviews or objective data, meaning participants may have over- or under-reported their symptoms. Future research should seek to further investigate the relationship between work demands and mental health outcomes (including alcohol use) by using objective measures, such as actual work hours recorded, rather than self-reported levels of demands. Second, women were over-represented in the sample (74% of respondents), although the gender distribution was consistent with patterns observed in the legal profession, especially amongst lawyers aged 49 and younger and those working in-house (Urbis, 2023). Future studies should seek to recruit larger samples, including more men. Third, the sample included mainly lawyers who worked in large or medium firms rather than smaller firms, so for this reason the results may not be generalisable to lawyers working in smaller firms. Fourth, participants included lawyers from Australia, the United Kingdom, North America, New Zealand, and ‘other’ countries, with the majority of participants (88%) being from Australia. There are important cultural differences between these countries, including in relation to variables in this study such as alcohol use. For example, previous research has found significant population-level differences between Australia and the United States with regard to alcohol use, with alcohol use substantially higher in Australia (77%) than the United States (46%) (Teesson et al., 2006). Nationality was not controlled in the analyses in this study due to insufficient sample size, and so future studies should consider the impact of cultural influences when examining relationships between variables tested in this research. Fifth, although a broader conceptualisation of wellbeing was used in this study, no physical elements of wellbeing were included as outcomes, which is a gap in the field more broadly (Soon et al., 2023). Sixth, although this study was informed by the JD-R model, it focussed on one pathway through job demands and personal resources in particular, with a need for future studies to expand consideration of this framework. For example, job resources such as workplace support, autonomy, and feedback should be included (Demerouti et al., 2001). Finally, due to the cross-sectional nature of the study design, causal inferences cannot be drawn. For this reason, there may be alternative interpretations of the results. For example, high distress and burnout may impact the way in which a lawyer perceives their work demands, or their capacity to engage in mindful self-care, rather than vice versa. As such, future studies should utilise prospective designs to enable temporal aspects of relationships in this study to be tested. Furthermore, cultural and social norms within law firms could also be explored to better understand how these factors impact lawyers’ engagement in mindful self-care activities, their mental health and drinking habits. Further exploration of lawyers’ attitudes towards the various components of mindful self-care, including whether the impediments to engaging in self-care are practical or attitudinal, is also warranted.

## Conclusion

This study examined the relationship between workplace demands and mental health in lawyers. Furthermore, it examined whether mindful self-care indirectly explained these relationships, and the moderating role of obsessive and harmonious passion in this model. Results showed that higher work demands were associated with higher distress and burnout, but were unrelated to alcohol use. Mindful self-care indirectly explained these associations, suggesting job demands might deplete personal resources enabling self-care in lawyers, which is associated with greater distress and burnout. The results suggest that higher work demands may increase a lawyer’s vulnerability to poorer affective mental health outcomes, which could be due to their association with lower levels of self-care activities that are protective. In contrast, non-significant findings for alcohol use suggest additional factors warrant consideration, such as social and cultural norms. The findings support the need for employers of lawyers to continue to address the organisational, systemic, and cultural factors, in particular high demands, that contribute to the mental health vulnerability of lawyers and for future research to further examine these factors and protective pathways to promote wellbeing.
